# Community perspectives on the benefits and risks of technologically enhanced communicable disease surveillance systems: a report on four community juries

**DOI:** 10.1186/s12910-020-00474-6

**Published:** 2020-04-25

**Authors:** Chris Degeling, Stacy M. Carter, Antoine M. van Oijen, Jeremy McAnulty, Vitali Sintchenko, Annette Braunack-Mayer, Trent Yarwood, Jane Johnson, Gwendolyn L. Gilbert

**Affiliations:** 1 Australian Centre for Health Engagement, Evidence and Values, University of Wollongong, Wollongong, Australia; 2 School of Health and Society, University of Wollongong, Wollongong, Australia; 3 Molecular Horizons and the Illawarra Health and Medical Research Institute, University of Wollongong, Wollongong, Australia; 4 Health Protection NSW Health, Sydney, Australia; 5 The Centre for Infectious Diseases and Microbiology - Public Health, Westmead, Sydney, Australia; 6 Marie Bashir Institute for Infectious Disease and Biosecurity, The University of Sydney, Sydney, Australia; 7 Cairns and Hinterland Hospital and Health Service, Cairns, Australia; 8 Cairns Clinical School, James Cook University, Cairns, Australia; 9 Rural Clinical School, University of Queensland, Brisbane, Australia; 10 Sydney Health Ethics, School of Public Health, The University of Sydney, Sydney, Australia

**Keywords:** Social licence, Data-linkage, Infectious disease, Pathogenomics, Public health surveillance, Public deliberation

## Abstract

**Background:**

Outbreaks of infectious disease cause serious and costly health and social problems. Two new technologies – pathogen whole genome sequencing (WGS) and Big Data analytics – promise to improve our capacity to detect and control outbreaks earlier, saving lives and resources. However, routinely using these technologies to capture more detailed and specific personal information could be perceived as intrusive and a threat to privacy.

**Method:**

Four community juries were convened in two demographically different Sydney municipalities and two regional cities in New South Wales, Australia (western Sydney, Wollongong, Tamworth, eastern Sydney) to elicit the views of well-informed community members on the acceptability and legitimacy of:

- making pathogen WGS and linked administrative data available for public health research
- using this information in concert with data linkage and machine learning to enhance communicable disease surveillance systems

Fifty participants of diverse backgrounds, mixed genders and ages were recruited by random-digit-dialling and topic-blinded social-media advertising. Each jury was presented with balanced factual evidence supporting different expert perspectives on the potential benefits and costs of technologically enhanced public health research and communicable disease surveillance and given the opportunity to question experts.

**Results:**

Almost all jurors supported data linkage and WGS on routinely collected patient isolates for the purposes of public health research, provided standard de-identification practices were applied. However, allowing this information to be operationalised as a syndromic surveillance system was highly contentious with three juries voting in favour, and one against by narrow margins. For those in favour, support depended on several conditions related to system oversight and security being met. Those against were concerned about loss of privacy and did not trust Australian governments to run secure and effective systems.

**Conclusions:**

Participants across all four events strongly supported the introduction of data linkage and pathogenomics to public health research under current research governance structures. Combining pathogen WGS with event-based data surveillance systems, however, is likely to be controversial because of a lack of public trust, even when the potential public health benefits are clear. Any suggestion of private sector involvement or commercialisation of WGS or surveillance data was unanimously rejected.

## Background

Outbreaks of infectious diseases can cause significant and costly health and social problems [1–4]. In Australia (and internationally) communicable disease risks are monitored through three core public health activities: (i) outbreak investigations; (ii) disease surveillance; and, (iii) public health research [5]. They all rely on systematic data collection to direct health resources and initiate public health actions in response to communicable disease risks and outbreaks. Two new technologies - pathogen whole genome sequencing (WGS; pathogenomics) and ‘Big Data’ analytics - could greatly enhance communicable disease control [6]. WGS is the ultimate microbiological strain-typing method. Effective communicable disease prevention and control depends on the accurate identification and characterisation of the causal pathogen - including the strain involved [7–9]. Increasingly sophisticated strain-typing methods have been developed over the past 20 years but, until recently, they have been expensive, time-consuming and/or poorly discriminatory. WGS systems that are faster, cheaper and more informative are being introduced into public health laboratories [10–13]. Their integration into infectious disease diagnosis and management will dramatically improve the accuracy and speed of pathogen identification and biological risk prediction. WGS produce universally understood data expressed in the sequence of nucleotides which can be used to reconstruct transmission pathways, highlight missing cases, and, potentially, locate an individual at the time of exposure [14–16]. Pathogen WGS is already being used in public health surveillance systems in Australia [17] and elsewhere [18–22]. Because infectious disease risks are universal, there are global calls for equitable sharing of WGS-based surveillance data to help distribute the benefits [23]. The increasing availability of pathogen sequence data will provide new resources for communicable disease research, control and surveillance at local, national and global scales [24, 25].

Meanwhile, advances in informatics have revolutionised the scope, accessibility and speed of data collection and analysis. “Big Data” refers to the rapidly escalating volume, velocity and variety of data produced and stored; “Big Data analytics” refers to ‘the process of collecting, organising and analysing large datasets, to discover patterns and generate useful, actionable information’ [26]. Technological innovation that assists collection, collation and interpretation of health-related information could enhance the efficiency and accuracy of communicable disease surveillance and outbreak investigation [27–29]. Government service providers hold large administrative datasets, and masses of data are generated by everyday life, for example retail transactions and internet and mobile phone use. If pathogen WGS data were linked to administrative and other data – such as geospatial tracking in mobile phones (GPS) and social media use for example – and examined systematically through an automated system, this information could be mined to uncover epidemiological associations much faster than traditional approaches [30]. The algorithms that analyse surveillance data have evolved substantially over the last decade [31]; incorporating them and novel information sources into established systems should provide earlier warning, more accurate monitoring and less uncertainty during early stages of outbreaks [25, 29, 32].

Current systems in New South Wales (NSW), Australia (as in many other jurisdictions) collect selected communicable disease-related data without individual consent under the powers granted by the Public Health Act 2010 [33]. This is justified by the need to operationalise public health protection, but tempered by a requirement that data are used only for public health purposes and personal information is protected [34, 35]. The benefits of earlier outbreak detection and response are significant. More expedient and specific interventions can limit the health and socioeconomic impacts of infectious diseases, but surveillance has political, personal and ethical implications [36, 37]. Risks include the use of flawed methods (as illustrated by Google Flu Trends®, which proved to have poor reliability) [38–40]. A trade-off may be required between data accuracy and privacy. Outbreak prediction or modelling based on inadequate data can have devastating economic, health and social consequences, but collecting, holding and sharing personal health information is a threat to privacy, no matter how great the potential benefits [41]. For example, WGS pathogen “fingerprinting” linked to clinical, epidemiological and spatiotemporal data will reveal information normally regarded as private (e.g. where a person has been, who they were with and the type of activity involved) [42–44]. Digital epidemiology – population disease detection and monitoring using ‘Big Data’ – raises similar issues [45]. Currently in public health research, data are de-identified and so might be assumed to be anonymous. However, in the absence of strict security measures, reidentification may be possible; re-identification usually can be achieved using linked metadata and other data sources, and hacking remains an ever-present risk [46–48].

This project was initiated because of concerns about the potential privacy implications of using WGS technologies to characterise the relationship between microbial isolates taken from patients. Potential implications of human genome sequencing were outside of the scope of this study. Decisions, about what data should be collected and what action thresholds are used, are not just matters for experts [37]. To secure social licence, these systems must align with community values; this can be facilitated by structured public dialogue to develop ethically and legally defensible justification for their design and operation [6, 49]. In this paper, we report on four community juries convened to consider the acceptability and legitimacy of using new technologies to enhance public health research and communicable disease surveillance. Research suggests that while the public tacitly accepts public health surveillance, they are less consistently convinced of the benefits of the secondary use of data for medical research [50–54]. Our aim was to ascertain, by means of community juries, the conditions under which a well-informed citizenry would or would not accept integration of pathogen WGS and Big Data analytics into communicable disease control, and why.

## Methods

Community juries and other forms of ‘mini-publics’ are an established and appropriate method of incorporating public values and preferences into health policy decision-making [55]. Unlike focus groups and surveys, they involve information exchange and constructive dialogue between members of the public and experts, with adequate time for consideration [56, 57]. The process is based on deliberative democratic theory; it is like a legal proceeding, except that outputs are not binding, Jurors’ decisions or recommendations provide evidence for policymaking [58, 59]. Members of the general public are brought together to receive detailed factual information about, and deliberate on values-based considerations [60]. They are given opportunities for discussion with experts and time to deliberate, before being asked to come to a consensus or majority verdict [56]. The method assumes that people can think rationally, revise their opinions on the basis of evidence, and make defensible decisions when provided with appropriate conditions. Community juries have been used in Australia and elsewhere to consider, for example, the introduction of new health technologies and health resource prioritisation [60–63].

**Fig. 1:**
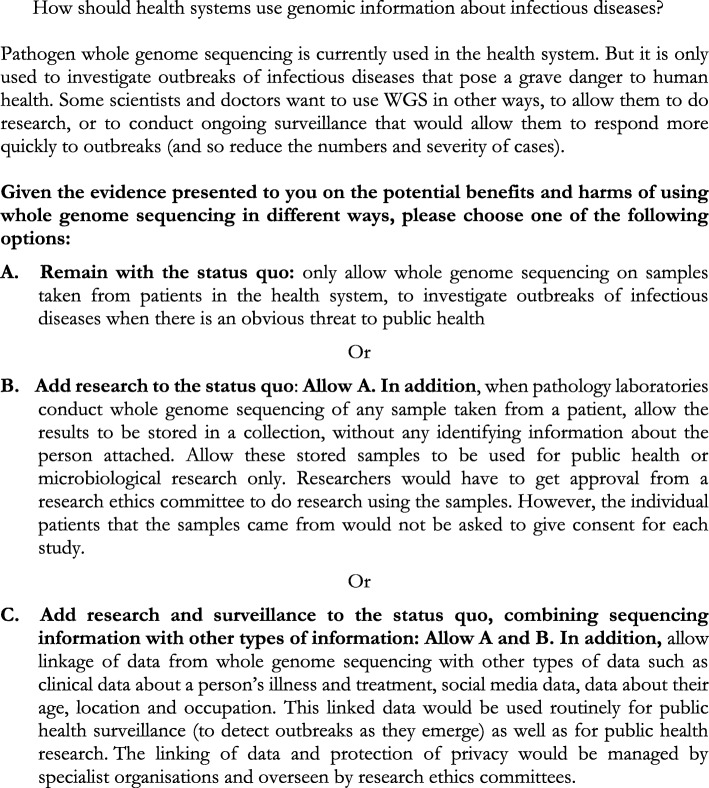
The question put to the community juries

Drawing on the outcomes of a preparatory Delphi process [49], we consulted a dozen relevant Australian policymakers and public health practitioners (including several State/Territory Chief Health Officers, directors of communicable disease branches and public health laboratories which employ WGS based surveillance of communicable diseases) to design the questions for the juries’ consideration (Fig. 1). Our study was approved by the Human Ethics Research Committee at the University of Wollongong.

To capture metropolitan and regional perspectives, we convened four community juries in NSW: Jury #1, western Sydney; Jury #2, Wollongong (south-eastern regional NSW); Jury #3, Tamworth (north-western regional NSW); and Jury #4, eastern Sydney. All were held over two days between November 2018 and March 2019. Western and eastern Sydney are large metropolitan communities of approximately 3 million people each; eastern Sydney is more urban and generally characterised by higher levels of social and economic advantage, whereas western Sydney is suburban and has greater ethnic and cultural diversity. Wollongong is the administrative centre for the Illawarra/Shoalhaven region (population 400,000 people), historically semi-rural, but now rapidly urbanising because of its coastal location and proximity to Sydney. Tamworth (population 200,000) is the administrative centre for the large and comparatively sparsely populated agricultural region of New England, in north-western NSW.

### Participants and recruitment

An independent professional research service (Taverner Research) was contracted to recruit participants at each study site, using randomly generated list-based samples of people who had previously volunteered to take part in research and topic-blinded social media advertising on Facebook. Jurors were selected from this pool of potential participants, based on representative socio-demographic characteristics (including gender and age); the final composition of each jury was determined by individual availability and eligibility, to ensure socioeconomic and cultural diversity within each jury. Fifty people were recruited across the four study settings (Table 1). All juries included participants with varied educational levels; the Tamworth jury was less socially and culturally diverse, reflecting the socio-demographic characteristics of the region. Jurors received a modest honorarium of $200 AUD per sitting day in recognition of their participation and contribution.

**Table 1:** Characteristics of Jury participants

	Jury 1 (*n* = 10^a^)	Jury 2 (*n* = 14)	Jury 3 (n = 14)	Jury 4 (*n* = 12^a^)
**Age (years)**				
18–34	2	4	6	3
35–54	4	7	6	5
> 55	3	3	2	3
**Gender**				
Male	4	6	7	4
Female	5	8	7	7
**Highest Educational Attainment**				
High School	3	3	3	4
Trade / Diploma	2	6	4	2
Bachelor Degree	3	3	5	4
Postgraduate Degree	1	2	2	1
**Socio-Economic Status of Place of Residence**^b^				
Low	3	2	2	2
Middle	5	8	12	4
High	2	4	0	5

^a^1 Participant of the 1st and 4th juries were unable to attend the second day because of illness

^b^Based on Socio-economic Index for Area (SEIFA

### Procedures

Each jury commenced with an orientation session to introduce the process and questions for consideration and obtain written consent. The content and scope of evidence presented changed between juries as shown in Table 2. Based on feedback from jurors, new presentations were added after Juries #1 and #2. Expert presentations were the same for Juries #3 and #4.

**Table 2:** Expert testimony provided to the community juries)

	Expertise	Expert area	Data provided	Events where presented
Talk 1	Molecular biology and diagnostic methods	Molecular Biosciences	• the basic characteristics and biological processes of microbial life and the microbiome • previous and current techniques used to identify and understand microbial processes and host/pathogen interactions • how microbial whole genome sequencing (WGS) works and the nature of the information it can provide	• Western Sydney (Jury#1) • Wollongong (Jury#2) • Tamworth (Jury#3) • Eastern Sydney (Jury#4)
Talk 2	Epidemiology and public health protection	Epidemiology & Communicable Disease Control	• the goals of outbreak investigations, infectious disease surveillance and public health research • why each of these domains of public health practice are important to population health • current systems, protocols, and legal controls employed in outbreak investigations, infectious disease surveillance and public health research in NSW, Australia	• Western Sydney (Jury#1) • Wollongong (Jury#2) • Tamworth (Jury#3) • Eastern Sydney (Jury#4)
Talk 3	Public health microbiology	Microbiology, Health Informatics & Infectious diseases	• the value of microbial genomic data and how WGS can be used to investigate, control and prevent infectious disease outbreaks • the potential public health benefits and ethical risks of using WGS to enhance communicable disease surveillance	• Western Sydney (Jury#1) • Wollongong (Jury#2) • Tamworth (Jury#3) • Eastern Sydney (Jury#4)
Talk 4	Data linkage, data security and communicable disease control practices	Infectious diseases, Data security, Big Data, & Data analytics	• potential benefits and risks of healthcare use of Big Data, data analytics and data linkage technologies • reasons to be concerned about data linkage, data security and data quality in communicable disease control and prevention practices	• Tamworth (Jury#3) • Eastern Sydney (Jury#4)
Talk 5	Bioethics, Public health ethics	Research Ethics & Health technology assessment	• current systems and best practices for health research involving data linkage (de-identification and linkage processes; models for participant consent) • ethical issues in using Big Data and Big Data analytics to enhance infectious disease control and prevention	• Wollongong (Jury#2) • Tamworth (Jury#3) • Eastern Sydney (Jury#4)

Public deliberation is enhanced by participants having access to evidence they need to make decisions that reflect their values and preferences [64, 65]. Therefore, as part of post-event evaluations, we asked jurors whether there were information gaps that should be addressed in subsequent juries and responded, iteratively, to the recommendations of each group. Several participants in Jury#1 told us they would have liked more information on current practices and protections in research involving data linkage and the use of Big Data analytics. This was provided, by adding a presentation on research ethics and data management (Talk 5 in Table 2). Feedback from participants of Jury #2 prompted inclusion of evidence focusing on risks to privacy of technologically enhanced surveillance, for Juries #3 and 4 (Talk 4 in Table 2), the latter indicated that they were satisfied with the scope and content of the information provided.

Testimony from our panel of experts was pre-recorded and shown to jurors as video presentations of about 30 min each. In consultation with an advisory group, experts were selected on the basis of their institutional roles, experience and expertise. They provided balanced and factual information supporting different perspectives on the processes, benefits and potential risks of using enhanced technology in communicable disease surveillance and research. Pre-recording ensured the evidence presented was standardised across juries. The experts’ bio-sketches and video presentations are available online [66]. After each presentation the expert was available by telephone or in person for question and answer sessions, facilitated by a researcher, which allowed jurors to clarify or question the evidence and opinions presented. Facilitation was positioned neutrally, and focused on promoting constructive dialogue and fair interaction amongst jurors.

For the first hour of the second day, the juries reflected on, discussed and debated the evidence, aided by a researcher acting as facilitator. As was the case in previous sessions, facilitation was explicitly neutral and focused on promoting constructive dialogue and fair interaction amongst jurors. Juries then deliberated for an hour, without researchers present, to reach a verdict on the questions posed. Their verdicts, reasoning and any dissenting views, were reported to the research team in a final feedback session. Our research and reporting processes for these community juries were cross-checked against the CJChecklist protocol [67].

### Data collection and analysis

The four juries are the units of analysis in this study. All jury deliberations and expert question and answer sessions were audio-recorded and transcribed. To track changes in positions held by individual jurors, participants completed an anonymous ballot three times - after considering evidence at the end of day one; after overnight reflection at the beginning of day two; and after their deliberation and delivery of the verdict at the end of day two. Jurors also completed an exit survey for the purposes of process evaluation, at the conclusion of each jury. During the final session a researcher recorded the verdict and reasons on a flipchart. Each point was reviewed by the jury to ensure accuracy. Transcripts were subsequently reviewed to identify and clarify key reasons why jurors supported or rejected the presented options. Results summarise jurors’ descriptions of the rationale and reasoning underpinning their responses.

## Results

Table 3 lists the outcomes of the final votes taken at all four juries. All of the juries supported the linkage of pathogen WGS data with administrative datasets for public health and microbiological research (Option B). The first three juries also supported application of Big Data analytics to linked pathogenomic and administrative datasets to enhance communicable disease surveillance (Option C) – noting that in Jury#3 this was only by a slim majority. The final jury held in eastern Sydney (Jury #4) voted against adding research and surveillance to the status quo (Option C) – once again by a slim majority.

**Table 3:** The final verdicts of the juries

	Jury#1	Jury#2	Jury#3	Jury#4
	Western Sydney (*n* = 9)	Wollongong (n = 14)	Tamworth (n = 14)	Eastern Sydney (*n* = 11)
**Option A. - Remain with the status quo:** only allow whole genome sequencing on samples taken from patients in the health system, to investigate outbreaks of infectious diseases when there is an obvious threat to public health	**0**	**0**	**1**	**0**
**Option B. - Add research status quo: Allow A. In addition**, when pathology laboratories conduct whole genome sequencing of any sample taken from a patient, allow the results to be stored in a collection, without any identifying information about the person attached. Allow these stored samples to be used for public health or microbiological research only. Researchers would have to get approval from a research ethics committee to do research using the samples. However, the individual patients that the samples came from would not be asked to give consent for each study.	**0**	**4**	**5**	**7**
**Option C. Add research and surveillance to the status quo, combining sequencing information with other types of information: Allow A and B. In addition,** allow linkage of data from whole genome sequencing with other types of data such as clinical data about a person’s illness and treatment, social media data, data about their age, location and occupation. This linked data would be used routinely for public health surveillance (to detect outbreaks as they emerge) as well as for public health research. The linking of data and protection of privacy would be managed by specialist organisations and overseen by research ethics committees.	**9**	**10**	**8**	**4**

Table 4 show the balance of the vote changed during the course of each jury with votes shifting between options A, B and C after provision of the evidence, overnight and after deliberation. Support for adding research and surveillance to the status quo (Option C) increased after participants had deliberated, but not enough in Jury #4 for this to become the majority position. Notably, all jurors supported current systems (Option A), such that there were no concerns or conditions stipulated about the use of pathogen WGS, administrative or publicly available social media data, during outbreak investigations and response. However, the support of jurors for using these technologies to enhance research (Option B) and research and surveillance (Option C) was contingent on several conditions being met. In what follows we describe the reasons jurors gave for supporting or rejecting a particular position, including any qualifying conditions. We then elaborate on the key points of consensus and disagreement between the juries.

**Table 4:** Votes at different time-points during jury proceedings

	CJ #1 (West Sydney)			CJ #2 (Wollongong)			CJ #3 (Tamworth)			CJ #4 (West Sydney)		
	OPTION A. Remain status quo	OPTION B. Add research	OPTION C. Add research & surveillance	OPTION A. Remain status quo	OPTION B. Add research	OPTION C. Add research & surveillance	OPTION A. Remain status quo	OPTION B. Add research	OPTION C. Add research & surveillance	OPTION A. Remain status quo	OPTION B. Add research	OPTION C. Add research & surveillance
#1 Saturday PM After evidence delivered	0	5	5	0	8	6	2	6	6	3	6	3
#2 Sunday AM After reflection overnight	0	4	6	0	9	5	1	4	9	3	5	3
#3 Sunday PM After deliberation	0	0	9^a^	0	4	10	1	5	8	0	7	4^a^

^a^Juror failed to attend on second day of proceedings because of illness

### Support for option B (adding research to the *status quo*)

As well as using WGS technologies for outbreak investigations, there was strong support for routine collection, storage and linkage of relevant pathogenomic and administrative datasets for use in public health and microbiological research. However, acceptance of option B depended on the following accompanying structures and controls being put in place.

### Voluntariness and participation

All 4 deliberative groups were of the view that active consent should not be required, for routine collection of WGS data of isolates from healthcare users in Australia. However, rather than allowing a consent waiver, a minority of jurors made the case that some people will have strong views against participation, which need to be accommodated. Having considered this requirement, each community jury independently came to the position that an ‘opt-out’ consent model was an appropriate compromise because it allowed individuals to withdraw, while leaving large enough datasets, by default, to maximise public health and research benefits. Various mechanisms for opting-out were discussed such as including a box on pathology request forms for patients to withhold consent for data-linkage and sharing, or establishing and raising public awareness about an online registry for people to indicate that they chose to opt-out of the system. None of the juries came to a definitive position as to how an opt-out system should be implemented, but all agreed that this consent model was most appropriate. This consensus was dependent on the Australian public being informed of the nature and purpose of any new use of patient data so people could opt out if they wished.

### Governance and funding

There was general agreement that the use of WGS data from patient isolates for research needs to be supported by a legislative framework. The National Health and Medical Research Council, which oversees the Human Research Ethics Committee system, was considered to be an appropriate institution to supervise pathogen WGS-related research activities. Jurors stressed that the system must be ‘not for profit’ and its governance and funding operated by a public body. There should be no selling of pathogenomic data to private interests nor direct corporate involvement in research. Public health laboratories, data custodians and linkage agents that support research, must be publicly funded to adequately maintain their integrity and sustainability and obviate a need for commercial support. At the same time research involving WGS data must have clear potential benefits to justify the costs of establishing and operating governance structures, with research results published in the public domain.

### Privacy, data linkage, data-sharing and security

Overall, none of the deliberative groups were overly concerned about potential privacy implications of pathogen WGS, if required, to characterise patient isolates. The technology was seen as a tool to create potentially useful information that could help save lives through the earlier identification of infectious disease outbreaks of public health importance. Jurors were far more concerned with how data generated were stored and shared and who could access it. Most important was that these data should be curated by organisations located in Australia. In general, jurors wanted only summary data to be shared internationally. Any linked data must be de-identified, protected by practices used for other government-operated administrative datasets, in Australia, and not publicly accessible, to reduce the risk of re-identification and protect privacy. Because WGS-related information could lead to adverse social, legal and actuarial consequences for individuals, jurors foresaw a need for legislation to reassure and protect the public from its misuse, including heavy penalties to deter hacking or leaking of data or unauthorised use by commercial (e.g. insurance or news media) organisations. In addition, individuals who are unwitting vectors of infection (e.g. of tuberculosis or gonorrhoea) must be indemnified, because of the risk of civil litigation, based on transmission events revealed by WGS.

The key reasons given by the one participant in Tamworth who disagreed with adding research to the status quo (Option B) were: (i) mistrust of security of current administrative data management systems; and (ii) concern about erosion of institutions in liberal democracies, and the potential for the goals of a data collection system to be re-oriented for authoritarian political processes. Other participants shared these concerns to varying degrees but ultimately voted to allow the use of pathogenomics and data linkage to enhance communicable disease research, as long as the conditions described above were met.

### Support for option C (adding research and big-data informed surveillance to the *status quo*)

As shown in Table 3 support for using the new technologies to develop and deploy Big Data- informed communicable disease surveillance systems was mixed. Support in each group for using these technologies for surveillance increased through deliberation (Table 4). But the level of support was weaker in the last two juries, probably due to their being provided with more detailed and specific evidence about the potential harm from use of Big Data analytics for surveillance. Nevertheless, all of the deliberative groups were excited by the potential public health value of Big Data analytics and there was qualified support for adding research and surveillance to the status quo (Option C), to improve health protection. But across the four groups, even when jurors were willing to support C, it was with strong conditions and without these conditions they would withdraw their support. As well as conditions specified for jurors’ acceptance of pathogenomic and data linkage in research (Option B), these extra conditions included the following.

### Voluntariness and participation

Linkage and centralisation of multiple datasets for routine surveillance made the deliberative groups more sensitive to the risks to privacy and liberty. They understood that a public health surveillance system based on application of Big Data analytics to linked and constantly updated datasets, would require at least wide public acceptance. Although jurors recognised that the public health value of option C would be reduced by incomplete participation, most felt that people should have a choice to decline participation if that was their preference. Consequently, all four juries were split on whether it should be an “opt in” or “opt out” system. This was a key sticking point for many participants; in the last two juries several participants who supported using pathogenomic and data linkage for public health research, told us they would have chosen to extend these systems to public health surveillance if it was explicitly organised as an opt in system. The same mechanisms for implementing the consent model were raised in these discussions, without coming to a resolution. Most jurors believed that decisions about consent were matters of public interest and should not be made solely by experts. Therefore, public awareness campaigns and consultation would be essential to ensure that people understood the choice and consequences of too few people participating.

### Governance and funding

The jurors in each deliberative group who supported the addition of Big Data analytics to automatically linked and updated datasets for surveillance (Option C) were of the view that such a system would require ministerial oversight and other checks and balances, such as regular monitoring and auditing by an independent statutory officer, analogous to an ombudsmen or privacy commissioner. All of the deliberative groups concluded that the transparency system to the public was important. The need for transparency extended to algorithms used to monitor data for the purposes of surveillance, including how algorithms work, how they were developed, by whom, and for what goal. This information and any changes must be publicly available in plain language. Because such a system could potentially be misused for political aims, all jurors thought the operating body and related data-linkage agencies, would need to be protected by robust supporting legislation to protect its regulatory independence. These supporting structures were regarded as important to make changing the purpose or practices of surveillance difficult and remove the role and remit of the operating organisation from politics. Finally, the intrusiveness of a system that tracked peoples’ day-to-day activities and could threaten their privacy meant that demonstrating effectiveness was important.

### Security, linkage and data-sharing

The jurors who supported technologically enhancing communicable disease surveillance (Option C) within each deliberative group were generally of the view that because commercial agencies already collect personal data for financial gain, allowing public health authorities to collect data for the public good was not that much of change. Nevertheless, there was disagreement within each group about the types of data that should be allowed to be perpetually linked for the purposes of syndromic surveillance. There was strong support for inclusion of pathogenomic and administrative health data in routine surveillance, but disagreement about extending the scope of surveillance by linking them to, for example, passively generated geospatial, retail and social media data. All participants believed that non-administrative datasets should be linked only when it was clearly “necessary” for health protection. There were differing positions on what thresholds or conditions should define “necessity” and on the balance of trade-offs between potential benefits and risks of including different types of data.

Participants who supported option C required clarity about data security, where and for how long data would be stored and who would have rightful access. Central to this were assurances of robust systems and penalties for non-compliance or misuse. Jurors believed that data should not be stored in perpetuity but should be deleted if no longer useful for actionable surveillance; they were also reluctant for surveillance data generated and stored in Australia to be shared with international agencies, unless absolutely necessary to manage a disease threat.

### Reasons not to extend to option C

The most important objection to option C was that, in the absence of a clear and present threat to public health, too much personal information would be available centrally, on the promise of future benefits. There were also concerns about the use of Big Data analytics to underpin surveillance; collection of so much data in one place would make dual use possible and create a ‘honey-pot’ for external and internal actors with malign intentions. Within each deliberative group there were jurors who took the position that because data security could not be guaranteed, caution is needed at the current stage of development; once established, such a system would be difficult to change.

Jurors who rejected option C believed that the current configuration of Australian public health and health protection systems were already meeting population needs, and did not require enhancement, unless demonstrable benefits could be realised, in the face of a major threat. They argued that Australian health authorities do not need to lead the development and application of Big Data analytics to surveillance, but could take advantage of other jurisdictions’ experience and improved technology as it emerges in other countries.

### Differences between juries

Support for option B was consistent across all juries. However, variations in strength of support for different options, between juries, suggests that the addition of more detailed information, about practices and governance of data-linkage processes (Jury #2 onwards) and potential risks of Big Data analytics (Jury #3 onwards), softened support for option C in later juries. If we regard the first two juries as pilot studies, the verdicts of the last two indicate that the application of Big Data analytics to perpetually linked datasets for communicable disease surveillance is likely to be highly controversial – especially if people do not fully understand its purpose or what is entailed in the establishment of such a system. Remembering that the task of jurors was to respond to the question and the evidence presented, through reflection, discussion and debate, the reasons given by Juries #1 and #2 for not supporting option C were also raised and discussed by Juries #3 and #4, but given greater weight by the latter.

The key differences between the groups were that more participants in Juries #1 and #2 saw the risks entailed by technologically enhanced communicable disease systems as being amenable to effective governance. In contrast, more participants in Juries #3 and #4 were concerned with the way in which data was stored, de-identified and protected. Differences in the socio-demographic characteristics of each deliberative group may explain this variation. But our impression during jury proceedings – which a review of the transcripts supports – was that exposure to detailed information and real examples demonstrating the current risks and limitations of data security arrangements, in Talk 4 (see reference [66] for details), appears to have made participants far less convinced that the potential public health benefits are worth the level of intrusion into how they conduct their day-to-day lives. Therefore, as deliberative groups, the final two juries were far more cautious about surrendering their privacy without clear evidence of effectiveness.

## Discussion

Our findings indicate that an informed public is likely to support the routine collection, linkage and use of administrative and pathogenomic data for the purposes of public health research. Participants were not overly troubled by the potential implications for people’s privacy of using WGS technologies to characterise the relationship between isolates taken from patients. Pathogen WGS was generally seen as being a powerful new test, the results of which could be managed under the same confidentiality protection that governed the sharing of other forms of personal health information by researchers and health professionals. Linking pathogen WGS data in a research context was also relatively uncontroversial because linkages between datasets were one-off bespoke events. Jurors were by and large content with the systems that govern data collection and sharing in health research in Australia.

However, juries made a strong distinction between research and surveillance such that the incorporation of these technologies into existing systems could cause controversy. While most jurors expressed excitement at the potential benefits of combing pathogen WGS with event-based data surveillance systems, there were also significant concerns. Most of this uneasiness was focused on the privacy risks and potential harms of the perpetual linkage and holding of multiple forms of data within a single system. For some participants, while the potential benefits of technologically enhancing communicable disease surveillance were understood, it was not obvious that the current system needed to be enhanced. Notably, the process of deliberation increased support for using these technologies to enhance communicable disease surveillance, because participants were able to articulate their concerns and think through different conditions that would need to be met for them to accept its implementation. However, as the weaker levels of support for Option C in the final two juries shows, the more people understand the risks, the less likely they are to support surveillance. Our results suggest that people intuitively react against being subjected to surveillance and, when they have more information, they become more hostile to implementation. But, if they talk with peers who are strong advocates of the benefits of surveillance, they are willing to soften their stance. Accordingly, most of the conditions imposed by jurors were designed to increase data protection and reduce the risk of data and privacy breaches, including limiting linkage to data held within health systems, and imposing strong governance conditions.

The outcomes of citizens’ juries held in the United Kingdom that considered data-linkage in the context of public health research are consistent with our findings. Most jurors at these UK events also wanted individuals to have the right to ‘opt out’ of data sharing arrangements and became less sceptical about health data sharing for public health research, as they became better informed of its benefits and risks [61]. They also wanted data to only be provided to organizations that could demonstrate that the primary goal for using the data was public benefit (either for new treatments or to improve existing services). For data-sharing at least our results are similar, as establishing an ‘opt-out’ system was preferred by most jurors in the current study for reasons of effectiveness, diversity and inclusion. Noting the citizens’ jury in the UK did not discuss the use of data-linkage in infectious disease surveillance, participants at our events became more sceptical about employing these technologies as more contextual information was added about the conditions of implementation. Evidence of potential harms was made less explicit for first 2 juries, and support for option C waned substantially once this evidence was formally introduced. Our findings highlight that people do not assess security enhancing technologies - such as surveillance - in abstract terms but in relation to the specific institutional and social context of implementation [68]. As shown by previous deliberative research [61, 69], people accept some loss of privacy for public benefits in specific circumstances.

Our findings highlight that surveillance systems are generally expected to be implemented in conditions consistent with strong public engagement and democratic processes, including clear public communication and strong transparency around data holding, use and analysis. Surveillance of various kinds is arguably becoming the norm, driven by both governments and corporations. In both democratic and non-democratic countries Closed Circuit TV (CCTV) is increasingly having facial recognition functions added [70]; internet usage is, as a Forbes journalist recently wrote, “designed to be a surveillance machine” rather than being “private by design” [71]. This is NOT occurring in line with the strict governance requirements that these juries recommended. The fact that people were more likely to reject surveillance when they understood more about it suggests that the existing forms of surveillance, were they to be more transparent, may also be rejected unless there was an extremely convincing public benefit- argument and effective independent oversight. The governance of public health systems tends to be more open than average and this project is an example of such a governance system being willing to engage with the public about the challenges it faces. There is an irony here. By seeking public input into surveillance system design, we could end up with tighter controls on a surveillance system that might be more likely to offer public benefit than many of the commercially oriented surveillance systems already in place. But, precisely because public health is about the common good and relies on trusting relationships between publics and governance systems, arguably public health authorities should uphold the highest possible standards to preserve trust. This process could however also be seen as a wakeup call for all the other state and non-state actors engaging in non-transparent surveillance.

The importance of evidence of organisational trustworthiness to the public acceptability of data-sharing and linkage has been shown in the UK [54, 61, 72] and internationally [73, 74]. Participants in the current study were initially resistant to allowing greater data-linkage in research but changed their position during the course of the event. But rather than simply trusting that the appropriate systems would be put in place, during their deliberations support among jurors very much depended on efforts to establish and sustain the trustworthiness of the system [64]. These included assurances and clarity about where the data goes and who would have access, the transparency of the operating and data-linking organisation, and the imposition of independent oversight and appropriate checks and balances. Any suggestion of private sector involvement or commercialisation of any of these systems was unanimously rejected across all four of the study settings. Public refusal of commercial involvement in the curation or analysis of data for public health purposes has been found elsewhere [51, 53, 73].

In principle, acceptance of new technologies and a willingness to trust their governing institutions is relatively easy to obtain. However, this underlying willingness does not necessarily translate into high levels of support for their implementation. The view that publics need to be educated so that they trust science and its governance is shifting. It is increasingly recognised that in order to establish the trustworthiness of new institutions, publics need to be actively involved in key policy decisions about the nature, content and scope of their activities [37, 64]. In their study of the governance of biobanks in Canada, O’Doherty and colleagues [75] identified several necessary conditions and institutional arrangements and qualities that work to establish trustworthy models of governance for new institutions. It is notable that, in their deliberations seeking to address the question asked of them, all four of these juries derived similar necessary conditions for the establishment of a new, potentially highly intrusive surveillance system. Table 5 is an adaptation of the criteria they developed that is applicable to other forms data-collection, aggregation and storage. Because O’Doherty and colleagues [75] established these principles in one context, and they were produced more or less *de-novo* by our deliberative groups in a different context, it is arguable that they capture something at the core of what citizens hold to be important. Incorporating highly structured processes for public deliberation and exchange, between mini-publics, practitioners and policy-makers, can support the development of knowledge that is essential to establishing [64, 76]:

(i) the agenda of what is important and needs to be considered when new technologies are implemented,
(ii) standards of practice that are transparent acceptable to the public, and, more broadly,
(iii) mechanisms that create and then maintain trustworthy systems of governance for public institutions.

**Table 5:** Necessary conditions for trustworthy governance of systems that link and aggregate personal administrative, medical and biological data

Characteristic	Example
Representativeness	• Consideration of the full range of individual and public interests
Accountability	• Ability to audit data use and management within operating organization; repercussions when violations occur
Transparency	• Overview of operations and decision making are open to scrutiny
Reflective Practice	• Regular review of operations and data use, including assessment of fit with original intent, approvals and consents
Sustainability	• Consideration of long-term financing and management

Table adapted from O’Doherty et al. [75]

As such the content and outcomes of our project and those conducted by the group in Canada [64, 75] could be a model for a form of participatory governance that enriches policies and practices by formalising a role for civil society in the governance of public institutions, especially those such as biobanking and data-linked research where surveillance activities can slide in by default.

### Study strengths and limitations

Community juries are a deliberative method that involve a process of iterative two-way exchange of information between members of the public and experts. By providing extensive information from a range of experts, and ensuring conditions for reasonable and extended debate, independent community juries elicit more considered judgements than other social research methods such as surveys or focus groups. The sample size is small, but this is necessary for high-quality deliberation. A possible limitation is that the evidence presented changed between Jury#1 and Jury#3 in response to participant feedback asking for more detailed evidence on the potential risks of technologically enhancing communicable disease surveillance systems. Because all four deliberative groups articulated similar positions and conditions for accepting system implementation, the new evidence changed the balance of the vote in the final verdict, and not the sets of reasons participants had for accepting or rejecting system implementation. A strength of this study was the quality and reputation of the experts who gave testimony, and the process by which they moderated one another’s presentations until all experts could accept that all views presented could be argued from the evidence.

## Conclusion

In conclusion, participants across all four events strongly supported the use of data linkage and pathogenomics for public health research, under current research governance structures, and for surveillance and outbreak investigation of diseases of public health significance. Combining pathogen WGS with event-based data surveillance systems, however, is likely to be controversial. Without clear mechanisms to establish and sustain the trustworthiness of a syndromic surveillance system, the broader public will be distrustful of its purposes and goals, even when the potential public health benefits are clear.

## Data Availability

No further data is available for analysis because of the conditions of ethics approval.

## Abbreviations

AUD: Australian Dollar
WGS: Whole genome sequencing

## References

[1] Kirk MD, Pires SM, Black RE, Caipo M, Crump JA, Devleesschauwer B (2015). World Health Organization estimates of the global and regional disease burden of 22 foodborne bacterial, protozoal, and viral diseases, 2010: a data synthesis. PLoS Med.

[2] Raviglione M, Sulis G. Tuberculosis 2015: burden, challenges and strategy for control and elimination. Infect Dis Rep. 2016;8(2):6570. 10.4081/idr.2016.6570. 10.4081/idr.2016.6570 PMC4927938 27403269

[3] Wang H, Wolock TM, Carter A, Nguyen G, Kyu HH, Gakidou E (2016). Estimates of global, regional, and national incidence, prevalence, and mortality of HIV, 1980–2015: the global burden of disease study 2015. Lancet HIV.

[4] Fan VY, Jamison DT, Summers LH (2018). Pandemic risk: how large are the expected losses?. Bull World Health Organ.

[5] Hawker J, Begg N, Reintjes R, Ekdahl K, Edeghere O, Steenbergen J (2018). Communicable disease control and health protection handbook.

[6] Gilbert GL, Degeling C, Johnson J. Communicable Disease Surveillance Ethics in the Age of Big Data and New Technology. Asian Bioethics Rev. 2019. 10.1007/s41649-019-00087-1 PMC7091643 32218872

[7] Sintchenko V, Iredell JR, Gilbert GL (2007). Pathogen profiling for disease management and surveillance. Nat Rev Microbiol.

[8] Gilbert GL, Selgelid M. Populations, patients, germs and genes – ethics of genomics and informatics in communicable disease control. In: Sintchenko V, editor: Infectious Disease Informatics. Basel: Springer; 2010. p. 397–418.

[9] Kwong JC, McCallum N, Sintchenko V, Howden BP (2015). Whole genome sequencing in clinical and public health microbiology. Pathology..

[10] Ashton PM, Nair S, Peters TM, Bale JA, Powell DG, Painset A (2016). Identification of Salmonella for public health surveillance using whole genome sequencing. PeerJ..

[11] Satta G, Atzeni A, McHugh TD (2017). Mycobacterium tuberculosis and whole genome sequencing: a practical guide and online tools available for the clinical microbiologist. Clin Microbiol Infect.

[12] Inns T, Ashton PM, Herrera-Leon S, Lighthill J, Foulkes S, Jombart T (2017). Prospective use of whole genome sequencing (WGS) detected a multi-country outbreak of Salmonella Enteritidis. Epidemiol Infect.

[13] Gurjav U, Outhred AC, Jelfs P, McCallum N, Wang Q, Hill-Cawthorne GA (2016). Whole genome sequencing demonstrates limited transmission within identified mycobacterium tuberculosis clusters in New South Wales, Australia. PLoS One.

[14] Koser CU, Holden MT, Ellington MJ, Cartwright EJ, Brown NM, Ogilvy-Stuart AL (2012). Rapid whole-genome sequencing for investigation of a neonatal MRSA outbreak. N Engl J Med.

[15] Arnold A, Witney AA, Vergnano S, Roche A, Cosgrove CA, Houston A (2016). XDR-TB transmission in London: case management and contact tracing investigation assisted by early whole genome sequencing. J Inf Secur.

[16] Dudas G, Carvalho LM, Bedford T, Tatem AJ, Baele G, Faria NR (2017). Virus genomes reveal factors that spread and sustained the Ebola epidemic. Nature..

[17] Laura F, PCG, Qinning W, Torsten S, Vitali S, Kathryn G (2018). Incorporating whole-genome sequencing into public health surveillance: lessons from prospective sequencing of Salmonella Typhimurium in Australia. Foodborne Pathog Dis.

[18] Ashton PM, Peters T, Ameh L, McAleer R, Petrie S, Nair S, et al. Whole genome sequencing for the retrospective investigation of an outbreak of Salmonella Typhimurium DT 8. PLoS Curr. 2015;7:ecurrents.outbreaks.2c05a47d292f376afc5a6fcdd8a7a3b6. 10.1371/currents.outbreaks.2c05a47d292f376afc5a6fcdd8a7a3b6 PMC4336196 25713745

[19] Byrne L, Fisher I, Peters T, Mather A, Thomson N, Rosner B (2014). A multi-country outbreak of Salmonella Newport gastroenteritis in Europe associated with watermelon from Brazil, confirmed by whole genome sequencing: October 2011 to January 2012. Euro Surveill.

[20] den Bakker HC, Allard MW, Bopp D, Brown EW, Fontana J, Iqbal Z (2014). Rapid whole-genome sequencing for surveillance of Salmonella enterica serovar enteritidis. Emerg Infect Dis.

[21] Hoffmann M, Luo Y, Monday SR, Gonzalez-Escalona N, Ottesen AR, Muruvanda T (2015). Tracing origins of the Salmonella Bareilly strain causing a food-borne outbreak in the United States. J Infect Dis.

[22] Inns T, Lane C, Peters T, Dallman T, Chatt C, McFarland N (2015). A multi-country Salmonella Enteritidis phage type 14b outbreak associated with eggs from a German producer:‘near real-time’application of whole genome sequencing and food chain investigations, United Kingdom, may to September 2014. Eurosurveillance..

[23] Edelstein M, Lee LM, Herten-Crabb A, Heymann DL, Harper DR (2018). Strengthening global public health surveillance through data and benefit sharing. Emerg Infect Dis.

[24] Besser JM, M'ikanatha N, Lynfield R, Van Beneden C, de Valk H (2013). Use of molecular epidemiology in infectious disease surveillance. Infectious disease surveillance.

[25] Gardy JL, Loman NJ (2018). Towards a genomics-informed, real-time, global pathogen surveillance system. Nat Rev Genet.

[26] Garattini C, RJ, Ausyah DN, Sartain F, Kozlakidis Z. Big data analytics, infectious diseases and associated ethical impacts. Philos Technol. 2017. 10.1007/s13347-017-0278-y PMC6451937 31024785

[27] Chowell G, Cleaton JM, Viboud C (2016). Elucidating transmission patterns from internet reports: Ebola and Middle East respiratory syndrome as case studies. J Infect Dis.

[28] Collier NH (2015). A Review of Web-based Epidemic Detection. The Politics of Surveillance and Response to Disease Outbreaks: The New Frontier for States and Non-state Actors..

[29] Wong ZSY, Zhou J, Zhang Q (2019). Artificial intelligence for infectious disease big data analytics. Infect Dis Health.

[30] Charles-Smith LE, Reynolds TL, Cameron MA, Conway M, Lau EH, Olsen JM (2015). Using social media for actionable disease surveillance and outbreak management: a systematic literature review. PLoS One.

[31] Yuan M, Boston-Fisher N, Luo Y, Verma A, Buckeridge DL (2019). A systematic review of aberration detection algorithms used in public health surveillance. J Biomed Inform.

[32] Bansal S, Chowell G, Simonsen L, Vespignani A, Viboud C (2016). Big data for infectious disease surveillance and modeling. J Infect Dis.

[33] Public Health Act 2010. No 127, New South Wales Government, Australia. 2010. https://www.legislation.nsw.gov.au/#/view/act/2010/127.

[34] Lee LM, Heilig CM, White A (2012). Ethical justification for conducting public health surveillance without patient consent. Am J Public Health.

[35] Rubel A (2012). Justifying public health surveillance: basic interests, unreasonable exercise, and privacy. Kennedy Institute Ethics J.

[36] Davies SE, Youde J (2012). The IHR (2005), disease surveillance, and the individual in Global Health politics. Int J Hum Rights.

[37] Fairchild AL, Haghdoost AA, Bayer R, Selgelid MJ, Dawson A, Saxena A (2017). Ethics of public health surveillance: new guidelines. Lancet Public Health.

[38] Ginsberg J, Mohebbi MH, Patel RS, Brammer L, Smolinski MS, Brilliant L (2009). Detecting influenza epidemics using search engine query data. Nature..

[39] Lazer D, Kennedy R, King G, Vespignani A (2014). Big data. The parable of Google flu: traps in big data analysis. Science..

[40] Lazer D, Kennedy S, et al. Wired. 2015.

[41] Fairchild AL, Bayer R (2004). Ethics and the conduct of public health surveillance. Science..

[42] Rothstein MA (2008). Keeping your genes private. Sci Am.

[43] Gilbert G, Selgelid M, Enemark C, Selgelid M (2012). Electronic surveillance for communicable disease prevention and control: health protection or a threat to privacy and automony. Ethics and security aspects of infectious disease control.

[44] Johnson SB, Parker M. The ethics of sequencing infectious disease pathogens for clinical and public health. Nat Rev Genetics. 2019;20(6):313–15. 10.1038/s41576-019-0109-3 30874624

[45] Vayena E, Salathé M, Madoff LC, Brownstein JS (2015). Ethical challenges of big data in public health. PLoS Comput Biol.

[46] Lubarsky B (2017). Re-identficiation of "anonymized data". Georgetown Law Technol Rev.

[47] Culnane C, Rubinstein BI, Teague V. Health data in an open world. arXiv preprint arXiv. 2017;171205627. https://arxiv.org/abs/1712.05627.

[48] El Emam K, Jonker E, Arbuckle L, Malin B (2011). A systematic review of re-identification attacks on health data. PLoS One.

[49] Degeling C, Johnson J, Gilbert GL (2019). Perspectives of Australian policy-makers on the potential benefits and risks of technologically enhanced communicable disease surveillance–a modified Delphi survey. Health Res Policy Syst.

[50] Kho ME, Duffett M, Willison DJ, Cook DJ, Brouwers MC (2009). Written informed consent and selection bias in observational studies using medical records: systematic review. Bmj..

[51] Hill EM, Turner EL, Martin RM, Donovan JL (2013). “Let’s get the best quality research we can”: public awareness and acceptance of consent to use existing data in health research: a systematic review and qualitative study. BMC Med Res Methodol.

[52] Whiddett R, Hunter I, Engelbrecht J, Handy J (2006). Patients’ attitudes towards sharing their health information. Int J Med Inform.

[53] Grande D, Mitra N, Shah A, Wan F, Asch DA (2013). Public preferences about secondary uses of electronic health information. JAMA Intern Med.

[54] Stockdale J, Cassell J, Ford E (2019). "Giving something back": A systematic review and ethical enquiry into public views on the use of patient data for research in the United Kingdom and the Republic of Ireland. Wellcome Open Res.

[55] Degeling C, Thomas R, Rychetnik L (2019). Citizens’ juries can bring public voices on overdiagnosis into policy making. BMJ..

[56] Street J, Duszynski K, Krawczyk S, Braunack-Mayer A (2014). The use of citizens' juries in health policy decision-making: a systematic review. Soc Sci Med.

[57] Degeling C, Atkinson PA, Williams RA, Cernat A, Sakshaug JW (2019). Deliberative Methods. SAGE Research Methods Foundation. In Press.

[58] Degeling C, Rychetnik L, Street J, Thomas R, Carter SM (2017). Influencing health policy through public deliberation: lessons learned from two decades of Citizens'/community juries. Soc Sci Med.

[59] Niessen C (2019). When citizen deliberation enters real politics: how politicians and stakeholders envision the place of a deliberative mini-public in political decision-making. Policy Sci.

[60] Degeling C, Carter S, Rychetnik L (2015). Which public and why deliberate? – a scoping review of public deliberation in public health and health policy research. Soc Sci Med.

[61] Tully MP, Bozentko K, Clement S, Hunn A, Hassan L, Norris R (2018). Investigating the Extent to Which Patients Should Control Access to Patient Records for Research: A Deliberative Process Using Citizens' Juries. J Med Internet Res.

[62] Newson A, De Lacey S, Dowling D, Murray S, Sue C, Thorburn DR (2019). Public attitudes towards novel reproductive technologies: a citizens’ jury on mitochondrial donation. Hum Reprod.

[63] Thomas R, Sims R, Beller E, Scott AM, Doust J, Le Couteur D (2019). An Australian community jury to consider case-finding for dementia: differences between informed community preferences and general practice guidelines. Health Expect.

[64] Burgess MM (2014). From ‘trust us’ to participatory governance: deliberative publics and science policy. Public Underst Sci.

[65] Dryzek J (2000). Deliberative democracy and beyond.

[66] Centre for Research Excellence in Emerging Infectious Disease (CREID). Community perspectives on the benefits and risks of technologically enhanced communicable disease surveillance systems 2019 [Available from: https://wp.me/P5i0Et-4o.

[67] Thomas R, Sims R, Degeling C, Street JM, Carter SM, Rychetnik L, et al. CJCheck Stage 1: development and testing of a checklist for reporting community juries – Delphi process and analysis of studies published in 1996–2015. Health Expectations. 2016. 10.1111/hex.12493. 10.1111/hex.12493 PMC5513001 27704684

[68] Pavone V, Esposti SD (2012). Public assessment of new surveillance-oriented security technologies: beyond the trade-off between privacy and security. Public Underst Sci.

[69] Parkin L, Paul C (2011). Public good, personal privacy: a citizens' deliberation about using medical information for pharmacoepidemiological research. J Epidemiol Community Health.

[70] Schippers B. Facial recognition: ten reasons you should be worried about the technology. The Conversation [Internet]. 2019. Available from: https://theconversation.com/facial-recognition-ten-reasons-you-should-be-worried-about-the-technology-122137.

[71] Toscano J. Google Has My Dead Grandpa’s Data And He Never Used The Internet. Forbes Now [Internet]. 2019. Available from: https://www.forbes.com/sites/joetoscano1/2019/09/03/google-has-my-dead-grandpas-data-and-he-never-used-the-internet/#73ea207b2b0c..

[72] Ipsos MORI. The One-Way Mirror: Public attitudes to commercial access to health data https://wellcome.ac.uk/sites/default/files/public-attitudes-to-commercial-access-to-health-data-wellcome-mar16.pdf Wellcome Trust; 2016.

[73] Damschroder LJ, Pritts JL, Neblo MA, Kalarickal RJ, Creswell JW, Hayward RA (2007). Patients, privacy and trust: patients’ willingness to allow researchers to access their medical records. Soc Sci Med.

[74] Willison DJ, Schwartz L, Abelson J, Charles C, Swinton M, Northrup D (2007). Alternatives to project-specific consent for access to personal information for Health Research: what is the opinion of the Canadian public?. J Am Med Inform Assoc.

[75] O’Doherty KC, Burgess MM, Edwards K, Gallagher RP, Hawkins AK, Kaye J (2011). From consent to institutions: designing adaptive governance for genomic biobanks. Soc Sci Med.

[76] Goodin RE (2017). The epistemic benefits of deliberative democracy. Policy Sci.

